# Audiological Outcomes and Associated Factors after Pediatric Cochlear Reimplantation

**DOI:** 10.3390/jcm11113148

**Published:** 2022-06-01

**Authors:** Fabian Blanc, Catherine Blanchet, Marielle Sicard, Fanny Merklen, Frederic Venail, Michel Mondain

**Affiliations:** 1 Department of Otolaryngology and Head and Neck Surgery, Gui de Chauliac Hospital, 80 Avenue Augustin-Fliche, 34090 Montpellier, France; fabian-blanc@chu-montpellier.fr (F.B.); c-blanchet@chu-montpellier.fr (C.B.); m-sicard@chu-montpellier.fr (M.S.); f-merklen@chu-montpellier.fr (F.M.); f-venail@chu-montpellier.fr (F.V.); 2 Institute for Neurosciences of Montpellier (INM), Institut National de la Santé et de la Recherche Médicale U1289, University of Montpellier, 80 Avenue Augustin-Fliche, BP 74103, CEDEX 5, 34091 Montpellier, France

**Keywords:** cochlear implant, reimplantation, audiological outcomes

## Abstract

Cochlear implants are the most common and successful sensory neuroprosthetic devices. However, reimplantation can be required for medical reasons, device failure, or technological upgrading. Resolving the problem driving the intervention and offering stable or better audiological results are the main challenges. We aimed to analyze the success rate of this intervention and to identify factors influencing speech perception recovery after reimplantation in the pediatric population. We retrospectively collected the causes and the outcomes of 67 consecutive reimplantations in one cochlear implant center over 30 years. Reimplantation resolved the cause without recurrence for 94% of patients. The etiology of deafness, time since implantation, indication of reimplantation, sex, and age did not influence word discrimination test scores in silence, 3 years after surgery. However, adherence to a speech rehabilitation program was statistically associated with gain in perception scores: +8.9% [−2.2; +31.0%] versus −19.0% [−47.5; −7.6%] if no or suboptimal rehabilitation was followed (*p* = 0.0037). Cochlear reimplantation in children is efficient and is associated with predictable improvement in speech perception, 3 years after intervention. However, good adherence to speech rehabilitation program is necessary and should be discussed with the patient and parents, especially for the indication of reimplantation for technological upgrading.

## 1. Introduction

Sensorineural hearing loss is the most common sensory deficit [1]. A cochlear implant (CI) is a neuroprosthetic device that enables the restoration of sound perception for patients receiving little or no benefit from hearing aids. In children with severe and profound sensorineural hearing loss, cochlear implantation is the reference rehabilitation [2,3]. Cochlear implantation is a safe and effective procedure, and CIs are considered the most reliable neuroprosthetic device. However, in 1.3 to 11.2% [4,5,6,7,8], reimplantation can be required. The causes include medical complications and device malfunctions. Device malfunctions can be separated into hard device failure (acute and complete loss of connection between the external and internal device with abnormal electrophysiological testing) and soft device failure (audiological performance decrement and exclusion of detectable hardware or software-related causes) [9,10]. More recently, the indication of reimplantation for technological upgrading of older implants has been discussed [11,12].

Offering stable or better audiological results after reimplantation is a major challenge. We hypothesized that the audiological outcomes may be influenced by several intrinsic and extrinsic factors: sex, age, etiology of deafness, timing of intervention, electrode array insertion, or the speech rehabilitation followed after reimplantation. 

In addition, few specific pediatric cohorts have been published regarding the percentage of success of this intervention. Cochlear reimplantation does not guarantee a resolution of the problem necessitating the intervention. Indeed, reimplantations sometimes fail to solve the medical problems or the suspected device malfunctions driving the intervention [6,9,13]. 

This study aimed to identify factors influencing speech perception recovery and evaluate the success rate of cochlear reimplantation in the pediatric population. 

## 2. Materials and Methods

We retrospectively collected the indications and the outcomes of 67 consecutive reimplantations in one CI center over 30 years (1989–2019). We included all consecutive cochlear reimplantations concerning patients that received their first CI before 18 years old. Overall, the reimplantation rate was 8.6% during the period (67/781 cochlear implantations). Cumulative survival was measured for each indication; subjects were censored yearly, and reimplantation dates were considered events (see Supplementary Figure S1).

The mean age at implantation was 4.8 +/− 3 years, ranging from 12 months to 15 years. Thirty-one boys and thirty-five girls with an age of 15.3 +/− 6.9 years underwent reimplantation. The time since initial implantation was 10.6 +/− 6.6 years, ranging from 3 months to 28 years. Etiologies of deafness are detailed in Table 1. The majority of etiology was genetic-related (46%). 

The primary outcome was the audiological performance, evaluated with open-set word testing in quiet of the phonetically balanced kindergarten words (PBK) [14]. The best scores obtained 1, 2, or 3 years after reimplantation were compared to the best results obtained before reimplantation. The consequence of reimplantation was thus expressed as a percentage decrease or increase in scores. Medical records were reviewed to identify the associated factors correlated with the evolution of word discrimination scores after reimplantation: sex, age, etiology of deafness, indication, best scores before reimplantation, time since the first implantation, difference in the angle of reinsertion of the electrode array (measured by cone-beam computed tomography according to Connor et al. [15]), and adherence to the speech rehabilitation program after re-implantation. Speech rehabilitation was systematically proposed to patients after cochlear reimplantation, on the same schedule than initial cochlear implantation. Participation in less than 50% of the speech rehabilitation sessions was considered “suboptimal” and represented 12% of the cohort.

No children with cochlear malformation underwent reimplantation in our cohort. Two children presented an enlarged vestibular aqueduct; complete reinsertion of the electrode array was possible in both cases.

Device failures were divided into hard failure 50% (*n* = 32), soft failure 30% (*n* = 20), and device failure in a context of head trauma 6% (*n* = 4). Medical indications included: infections in 7.5% (*n* = 5), 2 patients requiring deep brain stimulation to control severe dystonia (Mohr–Tranebjaerg syndrome), 1 patient presenting a displacement of the CI, and 3 patients presenting with non-auditory atypical symptoms during activation of the CI (headache, nausea, vomiting).

The success rate of reimplantation was assessed using specific criteria for each indication: better or stable audiological outcomes for the suspected device failures, recovery of the infection without recurrence of infections, and recovery of the non-auditive symptoms for the other causes. 

Prism 9.0.2 (GraphPad Software LLC, San Diego, CA, United States of America) was used for statistical analysis. Statistical differences in the audiological outcomes were compared using a non-parametric test for paired data (Wilcoxon’s rank test). The difference in the audiological outcomes as a function of the different putative associated factors were analyzed using a non-parametric test for unpaired data (Kruskal–Wallis and Mann–Whitney tests) whereas the correlation with quantitative associated factors were analyzed with the Spearman correlation coefficient. 

All subjects gave their informed consent for inclusion before they participated in the study. The study was conducted in accordance with the Declaration of Helsinki.

## 3. Results

### 3.1. Audiological Outcomes

The median words recognition test score was better after reimplantation than before: 78% [47–90%] versus 85% [65–92%] for the best score 3 years after reimplantation (median, 1st and 3rd quartile, Wilcoxon’s rank test for paired data, *p* = 0.006). The performances improved by over 10% in 46% (*n* = 23) of children, were similar (an increase or a decrease of less than 10% in scores between the implantation and the reimplantation) in 38% (*n* = 20), and showed a deterioration (decrease of more than 10%) in 16% (*n* = 7).

### 3.2. Factors Associated with Audiological Performance

We did not observe a statistically significant difference in the audiological outcomes regarding sex, etiology of deafness, or indication of reimplantation (Table 2). However, adherence to the speech rehabilitation program after the reimplantation was statistically associated with better audiological outcomes in the 3 years after reimplantation. 

**Figure 1:**
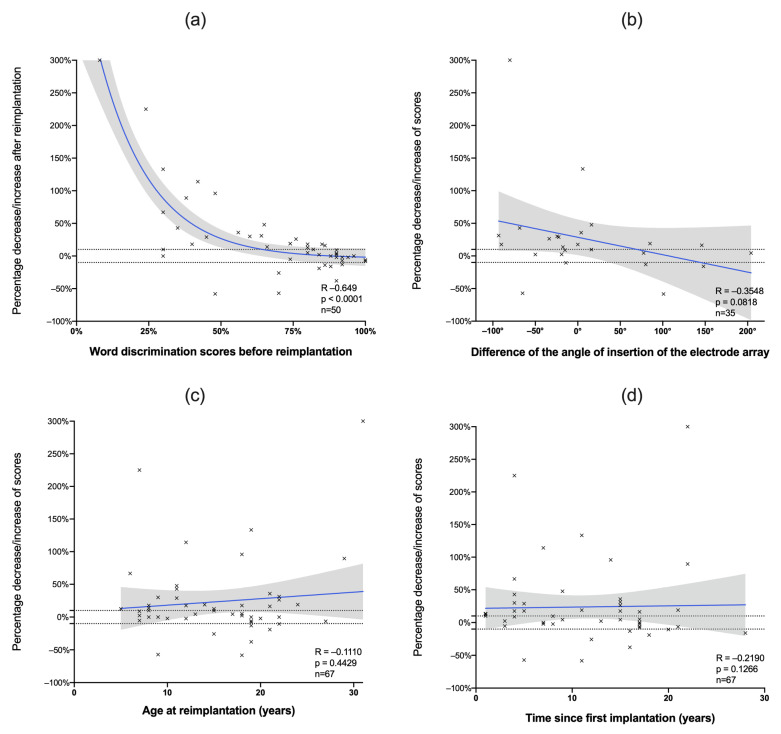
Correlation between the percentage increase or decrease in word discrimination and different factors: (**a**) Patients with low scores before reimplantation tend to have significantly increased scores in the 3 years after reimplantation, whereas patients with high scores tend to maintain audiological performance. The angle of insertion of the electrode array (**b**), the age at reimplantation (**c**), and the time since first implantation (**d**) were not correlated with the scores after reimplantation. Each patient cross represents a patient. Dotted lines: decrease or increase of 10% in word discrimination; blue line: simple linear regression; grey area: 95% confidence interval; R: Spearman coefficient of correlation.

The scores before reimplantation were correlated with the scores after reimplantation, and followed an exponential non-linear curve (Figure 1a, correlation of fit: 0.685). Indeed, the patients with low scores before reimplantation presented a greater gain than the patients with high scores. Conversely, the patients with high scores tended to have stable audiological performance after reimplantation. However, the angle of reinsertion (Figure 1b), the age at reimplantation (Figure 1c), and the time since the initial implantation (Figure 1d) were not statistically correlated with better audiological outcomes.

### 3.3. Success of Reimplantation

Reimplantation resolved the problem driving the intervention in 94% of patients. Four patients did not benefit from reimplantation (Table 3). The main hypothesis explaining these results were suspicion of auditory neuropathy spectrum disorder, scala vestibuli insertion of the electrode array, suboptimal speech rehabilitation, and initial diagnostic error. Patient 2 presented with ossification of the basal portion of the scala tympani. The reinsertion of the electrode array in the scala tympani was not possible despite several attempts of cochleostomies. The new electrode array was thus inserted in the scala vestibuli (complete insertion), but presumably had led to the decrease in auditory performances (−26%). For two other patients, partial reinsertion into the scala tympani occurred (the etiology was post-meningitis in one case, and unknown for the other case). Aside from these patients, complete reinsertion in the scala tympani was achievable in 96% of the cohort. For patient 3, the speech rehabilitation program was not followed because of the presence of severe tinnitus after reimplantation. The tinnitus was associated with anxiety and depression-like symptoms.

## 4. Discussion

The present study showed that cochlear reimplantation in children was efficient and associated with a predictable overall increase in audiological performances. Adherence to the speech rehabilitation program was associated with better audiological outcomes.

According to our results, word discrimination scores improved or were stable in 84% of patients; the scores showed poorer performance (i.e., decrease of more than 10%) in only 16% of patients. These results are in line with other reports in the literature: deterioration of audiological performances in only 2.9% for Rivas et al. [16], 37% for Henson et al. [17], and 10% for van der Marel et al. and Orús Dotú [18,19]. We did not observe any statistical correlation of these poorer results with sex, age, etiology of deafness, indication, time since the first implantation, and angle of reinsertion of the electrode array, consistent with other studies [20,21,22,23,24]. However, the audiological performance before reimplantation was found to be associated with the audiological outcomes: the patients with low scores tended to have a significant gain (up to +300%), whereas patients with high scores maintained these good performances after reimplantation (variation of less or more than 10%). This is an encouraging result, as patients with CI offering good performances seemed not to be at risk of significant decrement after reimplantation. This outcome favors the feasibility of replacing the old CI for technological upgrading without risking audiological performance decrement [12]. However, we observed that patients with suboptimal speech rehabilitation presented a median decrease of −19% in their performances. This finding is new in the context of cochlear reimplantation. It is in line with similar reported results after cochlear implantation [3]. In our center, the therapy consisted of teaching the child to use their residual hearing with optimal amplification (listening therapy) allowing the additional use of speechreading and/or natural gestures. The goal of these visual cues was to aid the child to understand the spoken language. The program also aimed to foster parental involvement, and to teach them how to create an optimal listening learning and language environment in everyday life, child’s daily routines, and play activities. Based on our findings, it seems that cochlear reimplantation should be associated with a thorough speech rehabilitation program to offer the best audiological outcomes after the intervention. Because of the retrospective design of our study, and the length of the cohort, it was difficult to quantify the speech rehabilitation program and analyze potential associated factors. We thus defined suboptimal rehabilitation as participation of less than 50% of the program. Non-adherence to the program (12% of the cohort) was because of the patient’s unwillingness, other intercurrent conditions (severe epilepsy, depression), or because of severe tinnitus in one case. It can be discussed that these factors by themselves could interfere with the audiological performances, and further studies need to be designed to understand the specific role of each factor. Moreover, the number of patients was small, and the calculation of a relative risk was not meaningful in this context because the confidence interval was too wide.

In our cohort, cochlear reimplantation presented a high success rate (94%). Only few studies are available in the literature on the pediatric population. One recent study observed a similar rate of 85% [6]. As in our cohort, the failure of cochlear reimplantation has revealed a central origin in some patients. They suspected an evolutive auditory neuropathy spectrum disorder in one case, and cochlear nerve hypoplasia in another case. In young children, the diagnosis of soft failure is often challenging. The absence of language development after implantation or the audiological performance decrement can evoke a soft failure [9]. However, other diagnoses can have the same presentation. In this context, the absence of language development may correspond to auditory neuropathy spectrum disorder, whereas audiological performance decrement may correspond to a degenerative central pathology. Finally, neurological delay or psychiatric conversion disorder are other possible final diagnoses if the reimplantation fails to restore the audiological performance [22]. Hence, several studies agree to consider that in these situations, as electrophysiological tests fail to reliably determine internal component functional status, the only option is to propose explantation–reimplantation [6,9,10].

In our study, another possible reason for failure in one case was the insertion of the electrode array in the scala vestibuli because of an ossified scala tympani. This patient’s score decrease by 26%. Audiological results after insertion into the scala vestibuli are reported to be worse, with an average score of word discrimination of 50% [25,26]. The insertion in the scala vestibuli could offer greater results if the scala media is not injured [27]. However, this technique presents a high risk of secondary degeneration of spiral ganglion neurons and remains a last chance option.

This study has several limitations. The retrospective design did not allow the analysis of certain data such as the quantification of the speech rehabilitation program. Moreover, it resulted in 47% of missing data, for the value of angle of insertion based on computed tomography. However, for our primary outcome, the audiological scores during 3 years after reimplantation were available for 75% of the cohort. Because of the indications of cochlear reimplantation, the cohort was also heterogeneous and of a relatively small size. However, its size remains average compared with the previously reported cohort [6,9,23]. Our long experience in cochlear implantation and the single-center design ensured that no major modification of the decision algorithm occurred during the study period. However, it may have introduced selection bias and may limit the possibility of generalizing these results.

## 5. Conclusions

Audiological performance improved after cochlear reimplantation in children. This intervention was highly efficient and tended to ensure stable performance in the patients with previously good audiological scores. Speech rehabilitation was an important factor associated with favorable audiological outcomes.

## Figures and Tables

**Table 1:** Etiologies of deafness.

Etiologies of Deafness	n	%
Genetic		
Nonsyndromic	19	28
Syndromic ^1^	12	18
Unknown	23	34
Meningitis	7	10
CMV	2	3
Labyrinthitis	2	3
Perinatal anoxia	1	2
Prematurity	1	2
Total	67	100

^1^ Including 6 patients with Usher syndrome.

**Table 2:** Percentage decrease or increase in word discrimination scores depending on sex, etiology, indication of reimplantation, and adherence to speech rehabilitation program after cochlear reimplantation (median and 1st and 3rd quartile).

		Percentage Decrease/Increase in Word Discrimination	*p*
Sex	Female	+7.50 [−3.02–28.7]	0.96
	Male	+9.32 [−1.63–26.4]	
Etiology	Unknown	+15.1 [3.89–33.4]	0.5
	Genetic nonsyndromic	0 [−2.21–4.44]	
	Genetic syndromic	+5.00 [−8.82–38.8]	
	Meningitis	+16.9 [3.75–20.9]	
	Other	+4.17 [−27.1–35.4]	
Indication of reimplantation	Hard failure	+12.5 [2.38–42.9]	0.052
	Soft failure	+10.0 [−1.09–27.6]	
	Medical indication	−13.0 [−31.7–4.75]	
	Head trauma	−2.13 [−3.77–3.81]	
Adherence to speech rehabilitation	Optimal	+8.89 [−2.15–31.0]	<0.01
	Suboptimal	−19.0 [−47.5–−7.63]	

**Table 3:** Description of patients receiving no benefit from the cochlear reimplantation. NSHL: non-sensory hearing loss; SHL: syndromic hearing loss.

	Etiology	Age	Time since Implantation	Indication	Surgical Findings	Word Discrimination Scores (after Reimplantation and Gain)	Comments
Patient 1	Perinatal anoxia ^1^	18 years	11 years	Soft failure	Complete insertion	20% (−58%)	Suspicion of evolutive auditory neuropathy
Patient 2	NSHL	15 years	12 years	Head trauma	Scala vestibuli insertion	52% (−26%)	Scala vestibuli insertion of the electrode array
Patient 3	SHL ^2^	21 years	18 years	Soft failure	Complete insertion	68% (−10%)	Suboptimal speech rehabilitation
Patient 4	NSHL	8 years	7 years	Medical reasons ^3^	Complete insertion	96% (+0%)	Pain after reimplantation remains stable—suspicion of migraine

^1^ Epilepsy and dysarthria; ^2^ Usher syndrome (type 1); ^3^ Pain around the processor.

## Data Availability

Not applicable.

## Supplementary Materials

The following supporting information can be downloaded at: https://www.mdpi.com/article/10.3390/jcm11113148/s1, Figure S1: Time after initial implantation (years).
