# Supplementary figures and images for: Audiological Outcomes and Associated Factors after Pediatric Cochlear Reimplantation
Source: J Clin Med. 2022 Jun 1;11(11):3148. doi: 10.3390/jcm11113148 (PMC9181352; doi:10.3390/jcm11113148)

# Survival

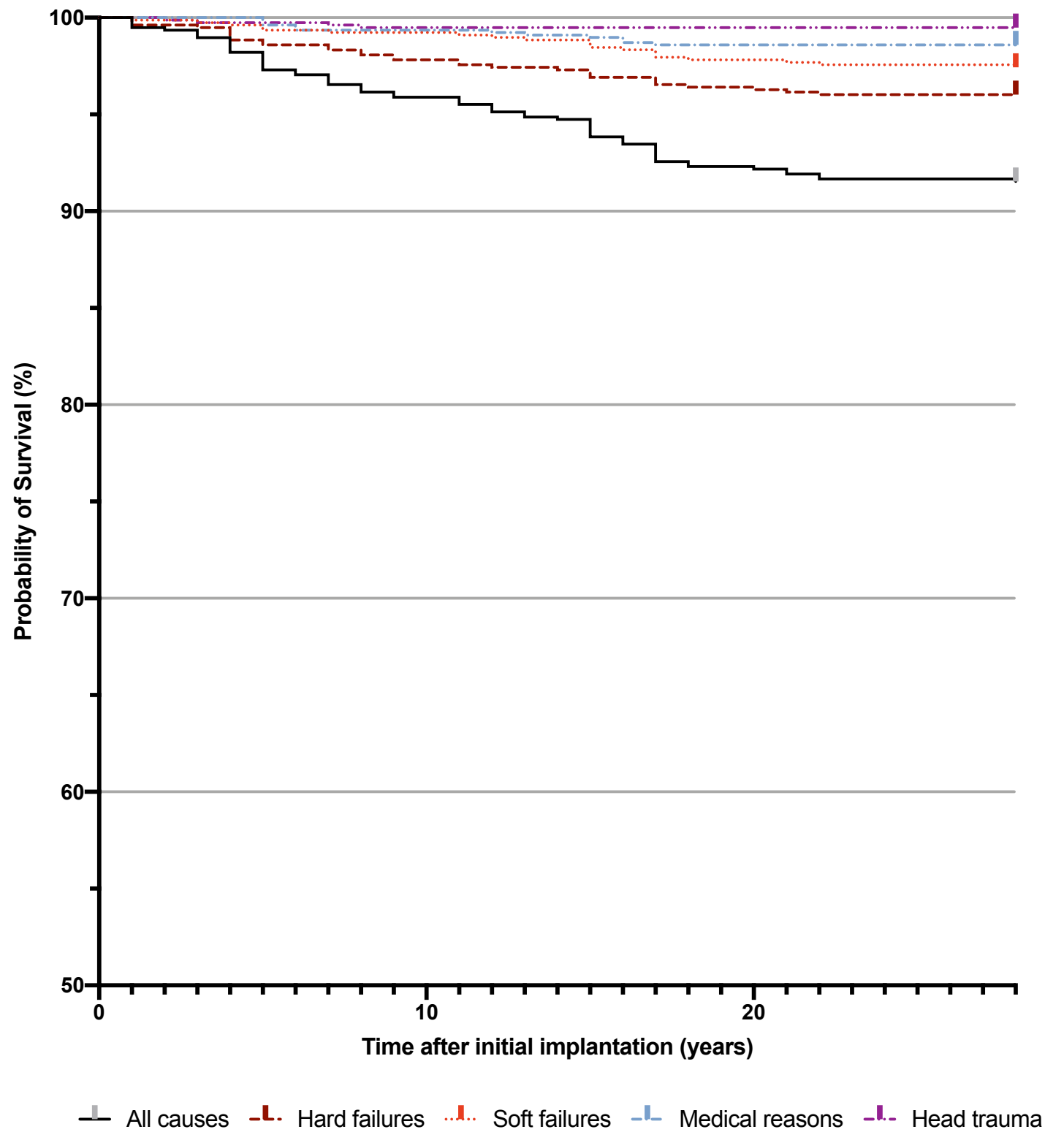

Supplement: Supplementary file 1 [file jcm-11-03148-s001.zip › Supplementary Figure S1.pdf]
